# Enhancing research careers: an example of a US national diversity-focused, grant-writing training and coaching experiment

**DOI:** 10.1186/s12919-017-0084-7

**Published:** 2017-12-04

**Authors:** Harlan P. Jones, Richard McGee, Anne Marie Weber-Main, Dedra S. Buchwald, Spero M. Manson, Jamboor K. Vishwanatha, Kolawole S. Okuyemi

**Affiliations:** 1 Center for Diversity and International Programs, University of North Texas Health Science Center, Fort Worth, TX 76107 USA; 2 Feinberg School of Medicine, Northwestern University, Chicago, IL 60611 USA; 3 Department of Medicine, University of Minnesota Medical School, Minneapolis, MN 55455 USA; 4 Initiative for Research and Education to Advance Community Health, Washington State University, Seattle, WA 98164 USA; 5 Centers for American Indian and Alaska Native Health, Colorado School of Public Health, University of Colorado Anschutz Medical Campus, Aurora, CO 80045 USA; 6 Department of Family and Preventive Medicine, University of Utah School of Medicine, Salt Lake City, UT 84108 USA

## Abstract

**Background and purpose:**

Preparing a successful research proposal is one of the most complex skills required of professional scientists, yet this skill is rarely if ever, taught. A major goal of the National Research Mentoring Network (NRMN) in the United States (U.S.) is to support the professional advancement of postdoctoral fellows and junior faculty from diverse populations by offering intensive coaching in the development of grant proposals early in their careers. This article highlights the National Institutes of Health’s (NIH) NRMN initiative to prepare diverse constituencies of early-stage biomedicine scientists for research careers by implementation of an evidence-based nationwide program of comprehensive grant writing and professional development.

**Program and key highlights:**

NRMN delivers four unique but complementary coaching models: the Proposal Preparation Program from the University of Minnesota (UMN); Grantwriters Coaching Groups from Northwestern University (NU); Grantwriting Uncovered: Maximizing Strategies, Help, Opportunities, Experiences from the University of Colorado Anschutz Medical Campus (UC) and Washington State University (WSU); and Steps Towards Academic Research from the University of North Texas Health Science Center (UNTHSC). Because these programs cater to scientists at different career stages, rather than employ a single approach, each is uniquely tailored to test its efficacy at the national level. The first two models prioritize scientists with reasonably well-developed research projects who are ready to write proposals for specific NIH research competitions. The other two models target postdoctoral fellows and early-career faculty who need more extensive guidance in proposal development plans. To achieve scalability, all programs also recruit faculty as Coaches-in-Training to learn approaches and acquire particular group facilitation skills required by each model.

**Implications:**

These efforts exemplify NRMN’s potential to enhance the career development of diverse trainees on a national scale, building research skills, competitiveness for obtaining faculty positions and capacities that will result in high quality research proposals from a diverse pool of applicants, thereby advancing innovations in science and diversifying the U.S. biomedical workforce.

## Introduction

Specific demographic groups defined by NIH are underrepresented in biomedical science careers in the U.S. [1, 2]. These groups include African Americans, Hispanics or Latinos, American Indians and Alaska Natives, Native Hawaiians and other Pacific Islanders, people with disabilities, and additional socially and economically disadvantaged populations. A high priority for NRMN is to enhance the number of faculty from diverse underrepresented groups who remain in the research workforce and advance in academic positions. This need is reflected in the latest statistics on the drop off in representation of URM populations at each of the key career stages. According to National Science Foundation’s report on Women, Minorities and Persons with Disabilities in Science and Engineering, 109,520 biological science bachelor’s degrees were awarded in the 2014. Hispanics or Latinos accounted for 11,552, 7663 to Black or African American, 269 to Native or Other Pacific Islander and 193 to American Indian or Alaska Native, compared to 63,320 Whites. A further discordance was reported in the number doctorate recipients in the biological sciences. The number of doctorates earned in the biological sciences in 2014 was 4067 for Whites, Hispanic or Latino 379, American Indian or Alaska Native 16, and 271 for Black or African American. Data on Native or Other Pacific Islander was either suppressed based on confidentiality or reliability [3]. An analysis of faculty diversity in top-funded U.S. Science, Technology, Engineering, Mathematics and Medical (STEMM) departments reveals that, in addition to the production of a very small diverse doctorate pool, even this limited diversity is not reflected in the faculty of biomedical and medical departments [4]. Moreover, faculty diversity by academic rank and race is further reflected in the disparity among underrepresented minority (URM) populations in the research workforce (Table 1).

**Table 1:** Percent of biology faculty by rank and race

	White	Asian	Black	Hispanic	American Indian
Assistant Professor	16.3	3.2	0.3	0.8	0
Associate Professor	19.4	1.8	0.3	0.3	0.1
Full Professor	53.2	3.1	0.4	0.8	0

A National Analysis of Minorities in Science and Engineering Faculties at Research Universities, Dr. Donna J. Nelson, Norman and OK. October, 2007

Career advancement for most research faculty is measured by their ability to obtain external funding support. This situation is particularly critical for tenure-track faculty, for whom the acquisition of NIH-level funding is often a requirement for promotion and tenure. One might argue therefore, that underrepresentation at the faculty level is likely further exacerbated by the discrepancy in rates of NIH funded research awards. In 2011, Ginther and colleagues confirmed a long-suspected disparity between racial minority groups and their White counterparts in terms of receipt of NIH research funding [5]. In 2016, Dr. Hannah Valentine, Chief Officer for Scientific Workforce Diversity at NIH, presented her “Analysis of African American Application Outcomes and Next Steps” at the 112th Meeting of the Advisory Committee to the Director of the NIH [6]. Dr. Valentine reviewed the recent success rates provided in the 2016 Office of Extramural Research Report, which found no improvement in success among African Americans compared to White applicants for NIH’s premier grant awards (e.g. 5-year research grants termed “R01”) between 2010 and 2015. During this period, African Americans represented only 1.5% of the total applicant pool, and among proposals submitted by African Americans, a lower percentage were discussed, scored, or resubmitted than among White applicants. This report identified a robust, sustained commitment to mentoring and coaching as a significant determinant of success in obtaining grant awards. Upholding and implementing accountability to this commitment on a national level is a primary goal of the NRMN.

One component of NRMN is the professional development of mentees and coaches. To this end, NRMN delivers four complementary coaching models: The Proposal Preparation Program (NRMN-P3) at the University of Minnesota (UMN), Grantwriters Coaching Groups at Northwestern University (NU), Grantwriting Uncovered: Maximizing Strategies, Help, Opportunities, Experiences (GUMSHOE) at the University of Colorado Anschutz Medical Campus (UC) and Washington State University (WSU), and Steps Towards Academic Research (NRMN-STAR) at the University of North Texas Health Science Center (UNTHSC). The following sections detail the creation and national dissemination of these intensive grant writing and coaching programs. Each is designed to provide diverse postdoctoral researchers and early-career faculty investigators with substantive innovative training and specialized mentorship to support the development of high-quality research proposals.

### The NRMN approach to grant-training

Crafting successful research proposals is among the most daunting and complicated skills to master, one that most investigators struggle to acquire and hone during the initial years of their academic appointments. Mentors can be valuable resources in modeling grant writing practices. However, many junior faculty do not have access to mentors with enough time to work with them to develop this essential competency; moreover, the degree to which mentors can or do teach grant writing skills is extremely variable.

In the past, NIH has tried to address this problem by providing grant writing workshops and other short-term professional development opportunities to early-career investigators. Many grant writing programs offer seminars; and some provide structured curricula spanning one or 2 days. However, the design and preparation of competitive research proposals require months of effort and iterative feedback from funded scientists, and grant writing skills often vary by career stage. This is particularly relevant for URM scientists who often have greater need for such development due to limited access where they are trained as well as poor resources to which they can connect [7–9]. Thus, acquisition of the necessary skills is subject to individual as well as environmental factors (e.g., grant writing experience, specialized consultation, access to instrumental resources, institutional support, adequate release time), and it requires a substantial investment of time. Accordingly, NRMN has intentionally built on the success four distinct but complementary programs designed to reach mentees at multiple levels of learning and career stages, offering mentees a tailored suite of programs. This unique and innovative model tests the ability to provide a multi-layered approach to professional development which can support a much larger number of mentees at each career stage and to a broader extent impact diverse constituencies that can support a sense of responsibility to a historically underrepresented demographic of the next generation of scientists.

Achieving a diversified biomedical research workforce requires grant writing and professional development activities with proven success on a national scale. A key innovation of the four NRMN programs is their integration of a “Coaches-in-Training” approach to expand their reach. There are very few studies of intensive interventions like career coaching in grant writing and professional development [10–12]. This feature involves “grantsmanship coaches,” a term used by NRMN to define a special subset of mentors: scientists with in-depth knowledge of their field and mentoring best practices as well as success in acquiring research awards. Notably, coaches must be motivated and committed to develop the skills of trainees from diverse populations. Coaches-in-Training refine their mentoring skills by participating in one of the four NRMN programs. They apply their well-practiced expertise in effective proposal writing (predominantly for NIH grant competitions) to provide tailored feedback on multiple iterations of mentees’ grant applications over a period of 2–12 months. Trainees also have access to advisors who can provide assistance related to their academic discipline and specialized research-based methodologies. NRMN expects new coaches to be ready either to lead their own coaching groups (with assistance as needed from the NRMN) or to work in collaboration with existing NRMN coaches. The overarching contribution is to make these coaching models accessible to postdoctoral fellows and early-stage investigators from diverse backgrounds, especially those whose home institutions do not offer intensive professional development. The NRMN coaching model is designed to complement, not to replace, the mentoring and guidance individuals may already receive in their departments and disciplines. However, an additional impact is also envisioned to provide the opportunity for faculty that become trained Coaches to return to their home institutions with the ability to create similar NRMN coaching and grant training.

### The specific training programs

Each of the four programs grew out of proven success in training early-stage investigators in grant writing and professional development at its home institution from which NRMN adopted a core of shared elements and unique practices from each in the creation of an inclusive approach to grant training. NIH supports a wide variety of designated research-related programs specified by project type, investigator initiated, budget allowed, length of research program and other additional criteria [13]. Two programs (NRMN-P3 at UMN and Grant Writers Coaching Groups at NU) enlist scientists with reasonably well-developed research projects who are ready to write K or R proposals, two of the main types of NIH research programs for funding. Skilled faculty coaches and peers identified by NRMN provide feedback on drafts of proposal sections over three to 5 months. The other two programs [14] recruit postdoctoral fellows and early-career faculty who need more extensive guidance in proposal development over 6–12 months (Table 2).

**Table 2:** NRMN program descriptions

Program	Duration	Intended audience	Unique approaches
Proposal Preparation Program (NRMN-P3)	2-day in-person kick off followed by 5 months of bi-weekly virtual meetings	Researchers who are ready to write at program start; preparing new or resubmission of a NIH-K or R-series proposal	Structured, writing intensive, small group with built-in mock study review
NRMN Grant Writers Coaching Group	2-day in-person kickoff followed by 4–5 months of virtual subgroup meetings scheduled as needed	Researchers who are ready to write at program start; preparing new or resubmission of a NIH-K or R-series proposal; special groups for R01-A1 submission are also available	Real-time feedback, strong emphasis on rhetorical patterns that are common to many NIH-style proposals
Grantwriting Uncovered (GUMSHOE)	3-day in-person kickoff followed by up to 6 months of virtual bi-weekly subgroup meetings	Researchers with none to minimal grant writing experience	Each cohort has a diverse, distinct population focus, Extensive engagement with NIH Grant Program Officials
Steps Toward Academic Research (STAR) Fellowship Program	Alternating in-person and virtual meetings over 12 months	Researchers with none to minimal grant writing experience	In addition to grantwriting coaching, training is also provided on professional development

The Professional Development Core of the National Research Mentoring Network (NRMN) offers four distinct but complementary programs to optimize participants’ career stage: *NRMN-P3* NRMN-Proposal Preparation Program, *NRMN NU* NU Grantwriters Coaching Groups, *GUMSHOE* Grantwriting Uncovered: Maximizing Strategies, Help, Opportunities, Experiences, *NRMN-STAR* NRMN-Steps Toward Academic Research

#### NRMN-proposal preparation program (NRMN-P3)

NRMN-P3 was adapted from a program at the UMN Medical School developed in 2007 by Dr. Richard King and Dr. Anne Marie Weber-Main. It originated as a 10-session in-person faculty development program in the Department of Medicine, and has since expanded to a thrice-yearly program, timed to coincide with standard NIH application deadlines. Enrollment is open to faculty investigators in the six schools and colleges within the university’s Academic Health Center. The structure and format of the Proposal Preparation Program **(**P3) are derived from traditional models of peer writing and review. A small cohort of 10–14 participants follows a prescribed schedule to write and revise drafts of major sections of their own proposals, and then convenes at regular intervals to receive feedback on their work-in-progress from three to five skilled coaches (senior investigators, scientific writing consultants) and from one another. Over the program’s lifetime, 20 cohorts have been trained, comprising more than 200 UMN investigators. Funding rates for these participants have ranged from approximately 20% to 40% per cohort – laudable outcomes in the context of significant budget reductions over the past decade at federal agencies and foundations. Given its success and popularity, the program has become a training requirement for all UMN faculty who receive significant funding for research career development from the university’s Clinical and Translational Science Institute KL2 Program and from the Building Interdisciplinary Research Careers in Women’s Health K12 Program [15, 16].

For the NRMN adaption of the UMN-P3, which was designed for researchers preparing their first major NIH research proposal, participants are expected to enter the program at the “ready to write” stage – that is, with reasonably well-developed scientific ideas, sufficient preliminary data, and the first draft of a Specific Aims page (which is a required section of all NIH proposals). This expectation helps to temper the false starts observed among participants who have not fully developed their planned approach or are still waiting for preliminary data to inform key aspects of their proposals. Participants are also asked to identify a local content mentor at or near their home institution (or a mentor from a previous training experience) who can offer discipline-specific input during proposal development.

The program begins with an intensive two-day, in-person workshop. To prepare for the workshop, participants are asked to critically read and comment on a draft of a Specific Aims page and a biosketch (which is used to evaluate PI expertise in review processes) submitted by another participant. On arrival, they immediately engage in the group review process. Program coaches and Coaches-in-Training facilitate group review and offer their own feedback. Afterward, participants attend individual coaching meetings to discuss their drafts in more detail. The workshop agenda is rounded out with didactic sessions (e.g., on the development of specific proposal sections, the use of graphics, and reader-friendly document design) and two interactive panel discussions. The first features recently funded early-stage investigators, who share their stories of persistence and lessons learned on the road to funding; the second features well-funded senior investigators, who offer their perspectives on how to interpret and respond to reviewer critiques in NIH Summary Statements.

NRMN-P3 training continues over the next four to 5 months in the form of seven virtual group review sessions conducted by videoconference. Participants deliver an oral critique of one of their peers’ drafts, drawing from guidance provided in writing rubrics and templates for Summary Statements. This process gives participants experience as reviewers while providing writing examples to emulate. It also fosters deeper connections within the cohort, creating the potential for future interdisciplinary and cross-institutional collaborations. Coaches provide oral critiques at each meeting, attending to each project’s scope, methodological rigor, and anticipated impact, as well as to its prose and presentation. Their explicit focus is helping trainees understand how to meet reviewers’ expectations for detail and clarity. Participants and coaches are encouraged to share more detailed written critiques or suggestions for revision by email. Peer review assignments are rotated within the group so that, over time, each participant can hear the perspectives of a range of reviewers. Participants’ local content mentors are also asked to attend two of these videoconferences so they can discern how the proposals might be perceived by researchers in disparate fields and assist participants with revisions between meetings. This schedule is ambitious, as it is designed to keep participants writing and revising well ahead of their submission deadlines. The program culminates in a formal review of completed drafts in the manner of an NIH study section. Reviewers for this event are solicited through recommendations by the participants and their local content mentors, or by inviting senior investigators who have applied to serve in this role through NRMN.

Coach training in NRMN-P3 occurs largely through real-time engagement with a cohort of mentees in collaboration with current coaches. The lead coaches strive to model desirable coaching behaviors. These include 1) bringing attention to common proposal criticisms that reviewers articulate during study section meetings; 2) prompting participants to include the specific details expected in the Approach and Training Plan sections of specific grant mechanisms or study designs, as these are often neglected by less experienced applicants; 3) identifying gaps in logic within the proposal narrative; 4) balancing honest criticism with appreciation for participants’ scientific ideas and writing progress; and 5) tailoring feedback to match participants’ skill level.

#### NU grant writers coaching groups

The conceptual and practical aspects of the NU Grant Writers Coaching Groups have been in development since the late 1990s. At that time, Dr. Richard McGee began looking for better ways to assist young investigators with grantwriting at the Mayo Clinic College of Medicine, which traditionally offered only day-long seminars. The core design elements of the Grant Writers Coaching Groups have been refined gradually since then, and in 2008 became the linchpin of research-related faculty development in the Feinberg School of Medicine at NU. Since 2008, more than approximately 300 junior faculty and postdoctoral fellows have engaged in some way with this novel group process. Writing groups begin every 4 months, and participants are encouraged to start even if they are unsure of their readiness to write. Over the years, about 40% of those who start in a group continue through proposal submission, and of those, about half are successful either with first or subsequent revised applications. Additional evidence of the effectiveness of this approach appears in the high rate of repeat participants and in word-of-mouth advertising from past participants to promote new enrollment. This model has also been successfully replicated and expanded in collaboration with the Association of American Medical Colleges through their annual Minority Faculty Career Development Seminar.

The NU model emphasizes sustained coaching over a three- to four-month period, offering trainees substantial practice in using the terminology and rhetorical patterns typically found in NIH-style proposals to convey key messages. A significant strength of this model is the ongoing work of the writing group, which engages in an intense and iterative process in real time, offering immediate, constructive feedback on successive drafts of each trainee’s proposal. The program begins with a one- to two-day session that provides an overview of the peer-review processes used by NIH, followed by an introduction to a series of online tools developed to guide grantwriters through each part of their proposals. The rest of the inaugural session is devoted to reviewing a draft Specific Aims page from each participant. Central to this model is oral feedback, led by the coach, which is not a critique but a review of the thought process triggered in the coach by each word, sentence, and paragraph in the draft. The dialogue and feedback that result are far richer than those typically achieved when a reviewer provides written comments. In addition, the group model enables participants other than the author of the draft under review to benefit from the process. They quickly begin to provide their own input as they practice the behavior modeled for them. The entire conversation is audio-recorded and sent to the author to use during revisions. After the initial in-person session, the writing group meets every one to 2 weeks by videoconference, employing the same process used in the in-person session. In most groups, at least three videoconferences are devoted to the Specific Aims page, a foundational component of proposals that must create a logical and scientific roadmap for the rest of the proposal. This focus enables simultaneous consideration of issues in prose construction, logical argumentation, and research design. A similar process continues for the Significance, Innovation, and Training components of other grant applications. Writing groups usually bring less attention to the Approach section unless they have the detailed scientific knowledge to give appropriate feedback. Over a three- to four-month period, this model allows for rich, ongoing discussions of the major sections of each trainee’s proposal. As needed, the group process can be further enriched by one-on-one sessions involving the coach and each trainee. In addition to facilitating the learning needed to master grantwriting, the group model encourages camaraderie among its members, potentially mitigating the feelings of isolation and insurmountable barriers that are often experienced by early-stage scientists.

As Coaches-in-Training, faculty with extensive experience writing and reviewing proposals rapidly develop the abilities needed to lead a group and employ oral and written feedback in an iterative process. Training in these skills is provided before any groups meet, so that new coaches can observe the approach modeled in an actual writing group during its initial in-person session. After the short experience of observing, new coaches are ready to adopt the model and take over their own groups right away. Additional guidance, as needed, is available to them from the model’s designer. In this way new coaches gain the knowledge and experience necessary to lead future cohorts. Among the most important facilitation skills that they acquire is how to balance peer-to-peer advice on a trainee’s proposal with expert critique. To date, more than 55 coaches have been trained in this model. They have led more than 50 groups comprising 190 postdoctoral researchers and junior faculty. By the end of 2016, 10–15 additional groups will be initiated with approximately 60 new participants and 12–15 new Coaches-in-Training.

### Grantwriting uncovered: maximizing strategies, help, opportunities, experiences (GUMSHOE)

Originating at the University of Colorado Anschutz Medical Campus (Dr. Spero M. Manson) and Washington State University (Dr. Dedra S. Buchwald), and initially offered in 2015, GUMSHOE springs from innovative best practices developed in the Native Investigators Development Program. The latter effort has been funded by NIH for the past 20 years, and has achieved remarkable success in increasing the number of American Indian and Alaska Native PhDs and MDs who obtain NIH research funding [17–19]. Since its inception, the program has retained 92% of applicants initially admitted, more than one-third of whom were tenured at their home institutions. It has also produced 48 graduates who have collectively generated more than 425 peer-reviewed publications and secured in excess of $100 million in NIH funding as principal investigators over the last 20 years.

GUMSHOE is offered twice per year. Each program cycle lasts 6 months, and each program cohort is limited to 25 participants who are supported by 8–10 coaches. To optimize participant/coach interactions and coordinate workshop emphases, each program cycle focuses on conducting NIH-sponsored research with a specific health disparities population or populations. These include 1) American Indians, Alaska Natives, Native Hawaiians, and other Pacific Islanders; 2) African Americans; 3) Hispanics/Latinos; 4) rural residents; and 5) other NIH-specified populations, as resources permit [2].

After they are selected for the program, through a multi-step process involving an application, letters of reference, a writing sample, and an interview, GUMSHOE participants are required to complete an online writing class. The curriculum involves completing exercises on writing the Specific Aims page of an NIH proposal; drafting and submitting their own Specific Aims page; and watching videos on the NIH process for proposal review. Mentees receive at least one round of extensive feedback regarding the Specific Aims submitted at the time of application, and must revise said aims accordingly prior to the initial meeting. Each participant is also assigned to a GUMSHOE coach by the two program directors (Dr. Manson and Dr. Buchwald).

After completing the online class, participants attend a three-day, in-person workshop that formally launches the program. The workshop introduces participants to one another and to their coaches, and includes didactic, experiential, and small-group work. Didactic elements focus on research support mechanisms, funding trends, key elements of application format and content, criteria for the multi-stage NIH review process, and common errors in preparation. Experiential components help participants select research questions that are amenable to investigation and that reflect the interests of potential sponsors as well as individual GUMSHOE participants. Once an appropriate question is chosen, workshop emphasis shifts to the process of crafting a well-articulated, persuasively written, logically consistent Specific Aims page as the cornerstone of the proposal. Small-group work involves frequent discussions that constructively critique each iteration of each participants’ Specific Aims in terms of clarity, logic, organization, and feasibility. Numerous examples of Specific Aims pages from previously funded applications are offered as models to emulate. Each participant then collaborates with his or her coach on establishing an individualized work schedule to draft, revise, and complete a full proposal over the remaining 6 months of the program cycle. Schedules typically include at least two 30-min telephone conferences per month.

Four key features of GUMSHOE distinguish this approach from other, similar offerings. First, during the three-day workshop, several senior methodologists circulate among participants and coaches to consult on features of participants’ study designs that are essential to addressing the research question proposed in the Specific Aims. Such features include the overall approach, sampling methods, and analytic plans. This early direction is especially effective in moving participants toward feasible, scientifically rigorous approaches and making proposal preparation more efficient. Second, the GUMSHOE workshop includes a 1.5 h interactive webinar that features a panel of three NIH Grant Program Officials (GPOs) who describe their respective journeys to service at NIH, review the nature and extent of their duties and responsibilities, and offer concrete strategies for when and how applicants should communicate with Program Officers. Their richly illustrated presentations are followed by wide-ranging question-and-answer sessions during which participants can introduce themselves and explore topics specific to their circumstances. Third, after each participant’s Specific Aims page is carefully vetted by the coaches, the program directors forward these pages – along with brief descriptions of participants’ interests, institutional affiliations, potential NIH Institutes or Centers, and preferred funding mechanisms – for review by a small, committed contingent of NIH grant program officers (GPOs). Their review attempts to ensure optimal alignment of the proposed work with the funding emphases of appropriate NIH units, and identifies responsible GPOs within those units. The GPOs, in turn, review and critique each Specific Aims page and offer a review of each proposal as it nears readiness for submission. Fourth, by focusing on specific populations of interest during each offering, GUMSHOE builds learning communities that transcend the program itself. Mentees -- faced with similar challenges in terms of science, institutional experience, and social expectations – bond in ways beyond disciplinary affiliation and commitment to research careers. They enjoy an opportunity to share a sense of mission with others that reinforces their original commitments and offers membership in a reference group that is personally as well as professionally meaningful.

The peer-to-peer learning process engendered during the GUMSHOE workshop is sustained over the ensuing 6 months through a web-based system that allows participants to post their completed application components (e.g., Specific Aims, Background and Significance, Innovation, Approach, Human Subjects, Facilities) for review and comment by their counterparts in the same cohort. In this way, learning moves beyond the coach/trainee dyad to include insights shared among participants. This process speeds the discovery of revised rules and emerging funding emphases. The web-based system also provides access to an array of resources relevant to the preparation of competitive applications, including examples of biosketches, proposals funded by various mechanisms, NIH Summary Statements, and follow-up responses.

To date, 30 coaches representing 19 different disciplines have mentored 89 NRMN mentees in the GUMSHOE program. All coaches are experienced NIH-funded investigators. Their training is accomplished through direct participation in the three-day workshop. It emphasizes modeling and frequent debriefing, combined with analysis of participant progress and barriers to success.

### NRMN-steps toward academic research (NRMN-STAR)

In 2005, UNTHSC established the Texas Center for Health Disparities (TCHD) to foster the relationships needed for effective health disparities research and to promote education and training for academic institutions. To achieve these ends, the TCHD Research Training and Education Core created the Steps Toward Academic Research (STAR) fellowship, which assembles a diverse group of early-stage faculty for training in professional skills and career advancement [20]. STAR emphasizes the conduct of health disparities research and the development of successful grant applications. The NRMN-STAR Coaching Group was subsequently modeled on STAR (Dr. Jamboor Vishwanatha and Dr. Harlan P. Jones, Program Directors). Its unique focus is the recruitment of faculty from historically under-resourced colleges and universities who need significantly more than a three- to six-month grant writing experience. Trainees of NRMN-STAR are selected based on their motivation and potential to develop a research project, with the understanding that many will require supplemental training. To meet this need, TCHD designed a 12-month curriculum. Its structure recognizes that junior faculty and postdoctoral fellows often find it difficult to accept a summer-long fellowship requiring an extended absence from home and family. NRMN-STAR therefore combines on-site professional development and education with distance learning, including online digital meetings and a resource repository. Throughout the year, participants focus on developing research proposals, with the goal of preparing a complete proposal to submit during the next NIH grant cycle.

The NRMN-STAR coaching model plays an integral role in facilitating grant writing and professional development. Coaches are established faculty from across the U.S. with extensive grant writing experience and interest in training early-stage researchers. Each coach is typically matched with two trainees based on research interest to form a coaching team. NRMN-STAR also utilizes “content coaches” to augment the grant writing process. Content coaches offer discipline-specific content expertise to trainees. They begin their training by attending a two-day kickoff meeting where they learn about the program curriculum and the principles of NRMN-STAR. Next, they engage in real time with the NRMN-STAR curriculum, as facilitated by the program directors. In addition to intensive coaching interactions within their teams, trainees and coaches benefit from activities in which the entire cohort forms a working group. This peer-to-peer experience builds a community of learners and fosters a broader exchange of best practices in grant writing and professional development. To date, NRMN-STAR has trained 11 coaches and 32 postdoctoral fellows and junior faculty.

## Conclusion and significant contributions

The skills required for grant writing at the postdoctoral and junior faculty levels are highly variable. Equally variable is the provision of institutional resources to support proposal development by early-stage investigators from underrepresented groups in biomedical, behavioral and social sciences. Therefore, professional development programs that extend beyond institutional borders are critically needed. NRMN’s significant contribution is the implementation of four intensive, complementary curricula in grantwriting and professional development that enables individualized, iterative feedback from successful senior researchers with demonstrated skills in working with minority investigators at different stages of their careers. The four curricula also help to develop a critical mass of grantwriting coaches with the requisite skills for this work. The Coaches-in-Training model implemented by NRMN prepares faculty to be effective grantwriting mentors, thereby providing a sustainable mechanism to deliver transformative professional development experiences to large numbers of underserved postdoctoral fellows and junior faculty.

### Current and anticipated outcomes

**Fig. 1:**
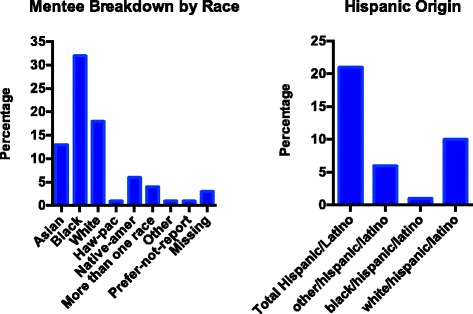
Race/Ethnicity of Mentees. Mentees self-reported their race/ethnicity defined as follows: White, Black, American Indian/Native Alaskan (AIAN), Asian, Hawaiian Pacific Islanders More than one race, Black/Hispanic/Latino, White/Hispanic/Latino, More than one race/Hispanic/Latino, other or did not report (Missing)

**Fig. 2:**
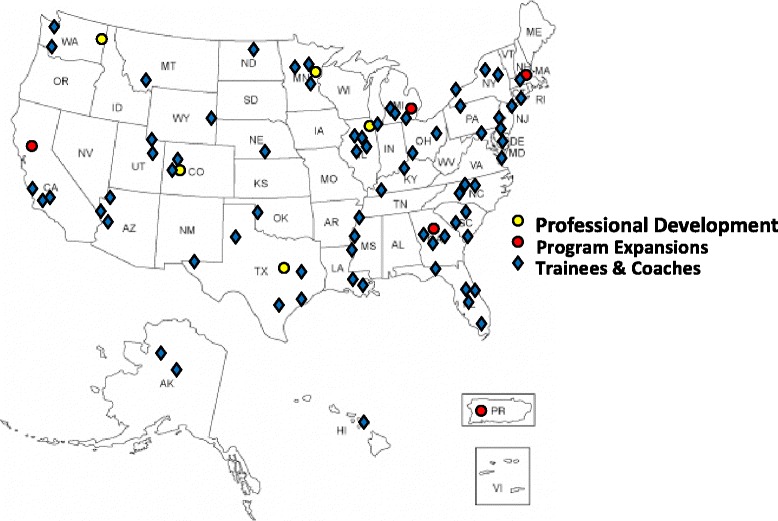
National Presence of Professional Development Core. NRMN’s Grantwriting and Professional Development Programs’ expansion. Original location of NRMN Grantwriting and Professional Development Program sites (yellow circle); new expansion sites (red circle) and location of Mentees and Coaches-In-Training (blue diamond) across the United States

A key outcome of NRMN’s approach to grant-training is to increase a sustainable architecture that will support and facilitate harnessing the collective expertise of successful scientists in the interest of accelerating the research career development of a diverse constituency of faculty. Considering that proposals are a key element of academic job applications, NRMN programs targeting postdoctoral fellows is one its impactful innovations. Although outcomes are premature, it is expected that such programs will also help URM scientists to be more successful in obtaining faculty positions. As an outcome, we expect to see an increase in the number and proportion of URMs progressing in their faculty career in part by receiving NIH research grant awards. Collectively, a diverse representation of URMs have participated as trainees across all programs including postdoctoral fellows and a growing diverse cadre of faculty Coaches-in-Training. Recruitment encompasses participation from partner minority-serving institutions, attendance at the Annual Biomedical Research Conference for Minority Students, Society for Advancing Chicanos/Hispanics and Native Americans in Science and fostering collaboration with scientific National professional societies. To date, African Americans (Blacks) and Hispanic/Latino populations comprised most of the participants followed by White and Asians. Collectively, Native-American and Hawaiian Pacific Islanders comprised made up 6% of the cohorts (Fig. 1). The NRMN Professional Development Core programs have successfully expanded from their original institutional settings by introducing five new locations to host one of NRMN’s grant writing programs. This has allowed NRMN to reach new participants trainees and train new coaches on a national scale (Fig. 2). Data on the efficacy of each program have begun to accrue, including numbers of proposals submitted, reviewed, resubmitted, and funded. In addition, baseline self-reported data is being collected from trainees providing value information of their grantwriting proficiency and confidence in persistence in biomedicine research. By the end of the first 5 years of funding, NRMN investigators will have accumulated substantial data to assess which program is most effective in which setting, and for which types of early-career trainees. The four programs are also learning best practices from each other and adding effective new practices to complement existing approaches. The support from NIH to expand four different models instead of collapsing them into a single program appears sound, as it promises to identify best practices for successful career development among minority researchers.

## Abbreviations

GUMSHOE: Grantwriting uncovered: maximizing strategies, help, opportunities, experiences
NIH: National Institutes of Health
NRMN: National research mentoring network
NU: Northwestern University
P3: Proposal preparation program
PDC: Professional development core
STAR: Steps toward academic research
UC: University of Colorado Anschutz Medical Campus
UMN: University of Minnesota
UNTHSC: University of North Texas Health Science Center
WSU: Washington State University
